# Diabetes Is Associated with Increased Autoreactivity of Mannan-Binding Lectin

**DOI:** 10.1155/2017/6368780

**Published:** 2017-02-28

**Authors:** Esben Axelgaard, Jakob Appel Østergaard, Steffen Thiel, Troels Krarup Hansen

**Affiliations:** ^1^Department of Biomedicine, Faculty of Health Sciences, Aarhus University, Wilhelm Meyer's Allé 4, Aarhus C, Aarhus, Denmark; ^2^Department of Endocrinology and Internal Medicine, Aarhus University Hospital and Department of Clinical Medicine, Faculty of Health, Aarhus University, Aarhus, Denmark; ^3^The Danish Diabetes Academy, Odense, Denmark

## Abstract

Mannan-binding lectin (MBL) has been reported to be involved in the pathophysiology of diabetic nephropathy. MBL is a pattern-recognition molecule of the innate immune system that initiates the lectin pathway of the complement system upon recognition of evolutionary conserved pathogen-associated molecular patterns or to altered self-tissue. Our group have previously shown direct effects of MBL on diabetes-induced kidney damage, and we hypothesized that MBL may cause autoactivation of the complement system via binding to neoepitopes induced by hyperglycemia. In the present study, we induced diabetes in MBL knockout mice and in wild type C57BL/6J mice by low-dose streptozotocin injection and measured blood glucose and urine albumin-to-creatinine ratio to monitor development of diabetes. After 24 weeks, fluorescently labelled recombinant MBL was injected intravenously in diabetic MBL knockout mice after which the distribution was investigated using in vivo fluorescence imaging. Mice were subjected to in vivo and ex vivo imaging 24 hours after injection. MBL was found to accumulate in the kidneys of diabetic mice as compared to healthy control mice (*p* < 0.0001). These findings support the hypothesis of a significant role of MBL and the complement system in the pathophysiology of diabetic nephropathy.

## 1. Introduction

The development of late vascular complications has been associated with chronic local inflammation. This is of major concern for patients with type 1 diabetes mellitus and constitutes a significant socioeconomic burden. The most common cause of renal failure in the Western world is diabetic nephropathy. Approximately 15–40% of all patients with type 1 or 2 diabetes will at some point need treatment for diabetic kidney diseases [1–4]. Several studies have linked the pattern-recognition molecule mannan-binding lectin (also known as mannose-binding lectin or MBL) of the complement system to the pathogenesis of diabetic kidney disease [5–9]. Recent studies in different mouse models have furthermore indicated a direct role of MBL in the pathogenesis of the renal changes seen during diabetes [10, 11]. Our group have recently demonstrated that MBL is found in the kidney during diabetes [12].

**Figure 1:**
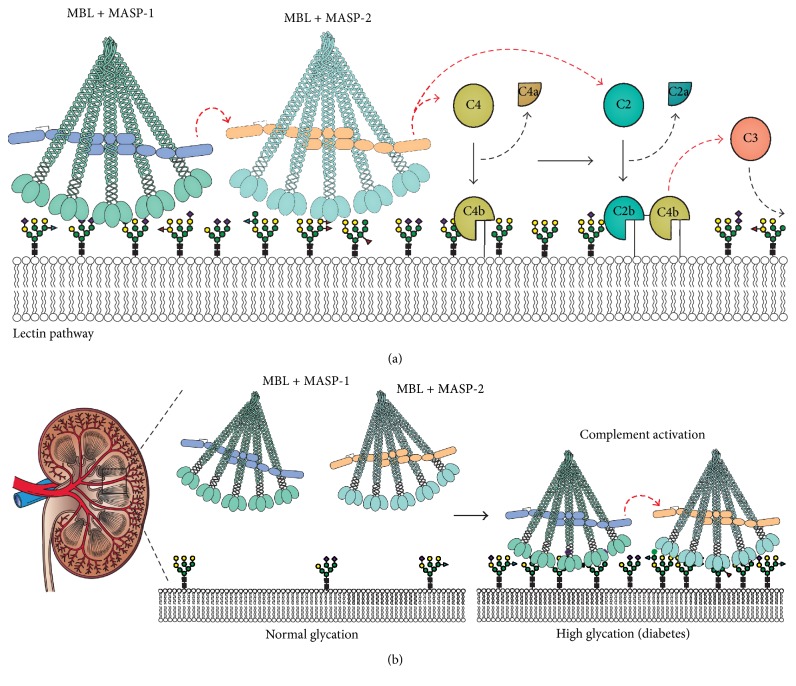
Overview of the lectin pathway of the complement system and study hypothesis. (a) Specific surface-sugar structures are recognized by MBL that leads to activation of the lectin pathway. The activation proceeds as an enzyme cascade with proteins acting as cofactors for each other thus generating opsonins for enhanced cellular recognition (C3b), anaphylatoxins that drive local inflammation (C3a and C5a), and terminally the membrane-attack complex (C5b-9) that mediates cell lysis. (b) Under normal glycemia MBL is unable to bind host cell-surfaces (left panel). However, during hyperglycemia the cellular surface may be altered, which mediates recognition by MBL and activation of the complement system.

The complement system constitutes an important part of the first line of defence of the innate immune system [13]. The system is generally divided into three different but interconnected pathways: the classical pathway, the lectin pathway, and the alternative pathway, all of which convey in a common lytic pathway. The complement cascade functions in the immediate responses to invading microorganisms. The system is based on complex interactions between more than 50 different surface-bound and soluble proteins. The common final outcome of complement activation is lysis of microorganisms and inflammation [14]. MBL is one of four pattern-recognition molecules of the lectin pathway that activates the complement system upon binding specific sugar ligands present on invading microorganisms and apoptotic/necrotic cells [15] (Figure 1(a)). Upon recognition of a specific ligand the MBL-associated serine protease 1 (MASP-1) undergoes structural rearrangement and thus initiates the pathway by activating a second MBL-associated serine protease (MASP-2). The active MASP-2 is able to cleave the complement proteins C4 and C4-bound C2, thereby generating a C3-convertase and initiating the complement cascade [16] (Figure 1(a)). Despite its obvious beneficial antimicrobial and homeostatic role, MBL has been associated with increased autoreactivity during several diseases [17–19]. The precise mechanism that links complement activation and diabetic nephropathy has not been clarified. The hyperglycemia seen during diabetes has been associated with increased glycations of proteins, endothelial structures, and cells [20]. Several studies have shown that there is a significant elevation in MBL levels in patients with type 1 diabetes [7]. Increased levels of MBL in combination with uncontrolled hyperglycemia may thus result in increased complement activation and contribute to and sustain local chronic inflammation and tissue damage. We hypothesize that long-term diabetes results in formation of nonenzymatic advanced glycation end-products (AGEs) and furthermore alters the enzymatic protein glycosylation pattern thereby enabling MBL to recognize such altered self-tissue and activate the lectin pathway (Figure 1(b)).

In the present study, we aimed to investigate the degree of MBL autoreactivity in a mouse models of type 1 diabetes using genetic MBL double-knockout (MBL/DKO) mice and noninvasive in vivo imaging followed by ex vivo imaging of the kidneys.

## 2. Research Design and Methods

### 2.1. Animals

For all experiments, we used 8-week-old age-matched male C57BL/6J BomTac wild type (WT) and/or MBL/DKO C57BL/6J BomTac mice (Taconic, Ry, Denmark) (MBL/DKO mouse strain lacks both murine isoforms of MBL, i.e., MBL-A and MBL-C). The development of the MBL/DKO strain has previously been described in detail [21]. In brief, the MBL/DKO mice were generated on a mixed Sv129EvSv and C57BL/6J background cross and later backcrossed onto the C57BL/6J background.

All animals had an initial body weight (BW) of 19–22 g and were housed 6 to 8 per cage with free access to autoclaved water and standard chow (Altromin 1324, Lage, Germany). Cages were placed in a room with a 12-hour artificial light cycle (7 a.m. to 7 p.m.), a constant temperature of 21 ± 1°C, and a humidity of 55 ± 5%. Animals showing signs of serious illness or more than 15% loss in body weight were excluded from the study. For all studies the BW and blood glucose (BG) were measured every 14 days. For the present study, we used two distinct animal groups for the various measurements: (1) estimation of glomerular volume and (2) IVIS study combined with albumin-to-creatinine (AcR) measurements. These groups are described in detail below under the relevant experimental methods. The IVIS Spectrum® is a noninvasive in vivo imaging system that allows for real time optical imaging of fluorescence and bioluminescence or monitoring of disease progression, cell trafficking, and gene expression. The study complied with the Danish regulations for care and use of laboratory animals.

### 2.2. Induction of Diabetes

Diabetes was induced by low-dose streptozotocin (STZ, Sigma-Aldrich Corp., St. Louis, MO, USA) protocol [22]. In brief, diabetes was induced by intraperitoneal (i.p.) injections with STZ for five consecutive days using a dose of 50 mg/kg BW dissolved in freshly prepared sodium citrate dihydrate buffer (10 mmol/L, pH 4.5) (Thermo Fischer Scientific, MA, USA). Nondiabetic control animals were injected with citrate dihydrate buffer only. Baseline BW and BG levels were determined before induction of diabetes. Prior to injection all animals were fasted for 4 h with access only to water. BG levels were measured after seven days. Poor responding animals were reinjected i.p. over two days with 50 mg STZ/kg BW. Animals were considered diabetic if having blood glucose levels >15 mmol/L. Nonresponding animals were excluded from the study.

### 2.3. Sample Collection

Blood samples and spot urine samples were collected every 14 days. During the study blood samples were drawn from the submandibular facial vein pouch and at the end of study from the retroorbital venous plexus followed by cervical dislocation. All blood samples were collected in serum collection tubes (Microvette® CB 300 *μ*L Sarstedt, Nümbrecht, Germany) followed by centrifugation at 1300*g* for 15 min at 22°C. Collected serum and spot urine samples were subsequently stored at −80°C until analytical use. Following in vivo imaging the kidneys were dissected under full anaesthesia and weighed followed by snap-freezing in liquid nitrogen in 1.5 mL CryoTubes® (Nunc, Sigma-Aldrich, Brøndby, Denmark). All snap-frozen kidneys were stored at −80°C. For estimation of glomerulus volume, the dissected kidneys were collected in 4% formaldehyde and stored at 4°C until use (see below).

### 2.4. Determination of Blood Glucose and Urinary Albumin-to-Creatinine Ratio (AcR)

BG was measured by tail-vein capillary blood using Accu-Chek® Aviva Nano (Roche, Mannheim, Germany). Urinary albumin and creatinine were measured after 24 weeks at study end in corresponding samples from male C57BL/6J MBL/DKO mice (*n* = 12) in the IVIS study group. Corresponding C57BL/6J WT male mice (*n* = 12) matched in age and degree of diabetes were included. Urine creatinine was measured in-house by isocratic Ultra-Performance Liquid Chromatography (UPLC) using a 50 mm × 2.1 mm Zorbax SCX300 column with a custom made in-front guard column (Agilent Technologies, Wilmington, DE, USA). In brief, 5 *μ*L urine was diluted in 100 *μ*L cold HPLC grade acetonitrile (AcN) containing 0.5% HPLC grade glacial acetic acid. To enable protein precipitation and harvest of a creatinine containing supernatant the urine samples were further diluted 10-fold in HPLC running solvent (5 mM sodium acetate ≥99.0% for HPLC, 4% HPLC glacial grade methanol, and 0.9% HPLC grade AcN) adjusted to pH 4.1 with ≥99.8% anhydrous glacial acetic acid (Merck, Hellerup, Denmark) and vortexed for 15 seconds to extract the creatinine. After storage for 15 min at −20°C and centrifugation at 10,000*g* for 10 min at 4°C, the supernatants were transferred to new eppendorf tubes and subsequently evaporated to dryness using a SpeedVac (Thermo Fischer Scientific, MA, USA) for 80 min. All samples were reconstituted with 25 *μ*L HPLC running solvent and briefly mixed to dissolve the residual creatinine. The 25 *μ*L was transferred to a 0.2 mL Skirted 96-well PCR autosampler plate (Thermo Fisher Scientific, MA, USA) and centrifuged at 3000*g* for 10 min at room temperature (RT) in a swing-bucket to avoid loading of any sample debris. Fractionation was performed with a constant column temperature of 45°C and a flow rate of 0.3 mL/min with a UV deuterium source detecting at 225 nm. Samples were run on the Acquity™ Ultra-Performance (UPLC) System (Waters, Milford, MA, USA) and analysed using the Empower™ Application Software. Samples were run in duplicate with three internal control urine samples (high, medium, and low in creatinine). The column was equilibrated for 60 min at the desired flow rate (0.3 mL/min) to ensure a steady baseline before loading the samples.

Urinary albumin concentration was measured in corresponding samples using a commercial Mouse Albumin ELISA quantification Kit (Bethyl laboratories, Inc., Montgomery, TX, USA), following the manufacturer's instructions, and AcR was calculated.

### 2.5. Estimation of Glomerular Volume

A second set of 8-week-old age-matched male C57BL/6JBomTac MBL/DKO (*n* = 21) were divided into two groups, where mice in the first group (*n* = 13) were treated with STZ (see above), while the mice in the second group (*n* = 8) were injected with buffer only. Blood samples were collected every 14 days from the submandibular facial vein pouch along with measurements of BG and weight gain as described above. Animals with signs of malcontent or illness (*n* = 3) were sacrificed and ignored. After 24 weeks, all mice were anesthetized with isoflurane and subsequently sacrificed by cervical dislocation. The mice were briefly stored on ice followed by dissection and weighing of both kidneys. The right kidneys were rinsed in PBS pH 7.4 and fixed in 20 mL 4% formaldehyde (CellStor Pot, CellPath, United Kingdom) followed by storage at 4°C. The left kidneys were snap-frozen in liquid nitrogen and stored at −80° until further use. The dehydration and paraffin embedding were carried out as follows. Each kidney was divided at the longitudinal axis and transferred to yellow paraffin embedding capsules and exposed to 70%, 96%, and 99% ethanol for 2-hour interval and finally with xylene overnight. The capsules were transferred to warm paraffin (60°C) and left to infiltrate for 2 hours before embedding. A metal capsule was filled with warm paraffin and stored on a warm plate of the embedding unit. The tissues were then placed in a sagittal cutting position in the metal mold and transferred briefly on a cold plate to fasten the tissues and prevent relocation before more paraffin is added. The capsules were placed on a cold plate and subsequently stored at 4°C until use. Each paraffin block containing one sagittal-cut kidney half was trimmed by 40 *μ*m on a rotatory microtome and matched kidney halves were placed on the same microscope glass slides and stained with Periodic Acid-Schiff (PAS) according to the manufacturer's instructions (VWR, Bie & Berntsen, Søborg, Denmark). Blinded estimation of glomerular volume was performed using a light microscope (LM) at 25x magnification based on Weibel's formula in Image Pro Plus software using 50 or more glomeruli per section per kidney [23].

### 2.6. Recombinant MBL

Recombinant MBL (rMBL) (Enzon Pharmaceuticals, FL, USA) was labelled with the Alexa Fluor® 680 near-IR reactive dye (AF680, cat# A200008) following the manufacturer's instructions. In brief, the AF680 dye was dissolved in dimethyl sulfoxide (DMSO) at 10 mg/mL and stored at −20°C until use. The rMBL was diluted to a final concentration of 1 mg/mL and incubated in darkness with the AF680 dye in a molar ratio of 1 : 20 for 1 h at RT on a rocking table. Unbound dye was removed from the protein solution by dialysis against 2 L PBS (137 mM NaCl, 2.7 mM KCl, 1.5 mM KH_2_PO_4_, and 8.1 mM Na_2_HPO_4_, pH 7.4); incubation was 2 × 2 h during the daytime and 1x overnight in SpectraPor dialysis tubes (Cole-Parmer, IL, USA). The tubing was preblocked in TBS/0.05% Tween (10 mM Tris-HCl, 140 mM NaCl, 15 mM sodium azide, and 0.05% v/v Tween 20, pH 7.4), followed by wash with Milli-Q® water (Merck Millipore, Life Science, MA, USA) to block residual binding sites, thus avoiding unspecific protein binding to the tube. The final protein concentration was determined using NanoDrop1000 by correcting for the absorbance of the dye.

### 2.7. Test of Function of the AF680 Labelled rMBL

FluoroNunc MaxiSorp microtiter plates (Thermo Fisher Scientific, MA, USA) were coated with 10 *μ*g/mL mannan in carbonate coating buffer (15 mM Na_2_CO_3_, 35 mM, NaHCO_3_, and 15 mM NaN_3_, pH 9.6). The time-resolved immunofluorometric (TRIFMA) assay for MBL has previously been described in detail [24]. The AF680 labelled rMBL and unlabelled rMBL were incubated directly on mannan-coated plates in either TBS/0.05% Tween/Ca^2+^ or TBS/0.05% Tween/10 mM EDTA (Figure 3(a)). In parallel, the AF680 rMBL and unlabelled rMBL were first incubated in a different setup with various concentrations of either mannose (expected inhibitory) or galactose (expected noninhibitory) (0, 6.25, 12.5, 25, 50, and 100 mM) in TBS/0.05% Tween/Ca^2+^ for 1 h at RT on rotation (Figure 3(b)). The samples were then incubated on mannan-coated plates overnight at 4°C and developed as follows. Wells were washed three times in TBS/0.05% Tween/Ca^2+^ and incubated with a biotin-labelled anti-MBL antibody for 2 h at RT. The plates were then washed three times in TBS/0.05%Tween/Ca^2+^ followed by incubation with Eu^3+^-labelled streptavidin for 1 h at RT. After another three washes 200 *μ*L enhancement solution was added to the wells. Fluorescence was detected on a fluorometer (Victor^5^, Perkin Elmer, Waltham, MA, USA). Possible compromising effects of labelling on the oligomerization of rMBL were examined by protein silver staining as described below. Data is given as percentage of bound MBL where mean values obtained in the presence of galactose were defined as 100% bound and subsequent samples were calculated according to these. The experimental data is an average of three individual experiments.

### 2.8. SDS-PAGE and Protein Staining

Sodium dodecyl sulphate polyacrylamide gel electrophorese (SDS-PAGE) and silver staining of bound proteins were carried out as follows. AF680 labelled rMBL or rMBL (0.5 *μ*g/mL pr. well) were diluted in SDS-PAGE sample buffer (30 mM Tris-HCl, 10% (v/v) glycerol, 8 M urea, 3% (w/v) SDS, and 0.1% (w/v) bromophenol blue, pH 8.9) and separated on a 4–15% Criterion™ TGX™ precast Tris/Glycine gel (Bio-Rad Laboratories Inc., US) for 2.5 h at 110 volts. Proteins in the gel were visualized by protein staining as previously described [25].

### 2.9. IVIS Spectrum Study Design

The C57BL/6J MBL/DKO mice (*n* = 24) were initially randomised into two groups: (1) diabetic (*n* = 12) and (2) nondiabetic control (*n* = 12). BG and BW were measured every 14 days during the study in addition to collection of blood and urine samples. STZ treated mice showed mean blood glucose levels above 15 mmol/L (mean of 16.6 mmol/L) after 28 days and were thus considered diabetic. After additional 24 weeks, the mice were further divided as follows: (1) diabetic + rMBL AF680 (*n* = 6), (2) diabetic + PBS (*n* = 6), (3) control + rMBL AF680 (*n* = 6), and (4) control + PBS (*n* = 6). The mice were injected intravenously (i.v.) with either 800 pmol/kg BW of rMBL AF680 (groups 1 and 3) or PBS buffer pH 7.4 (groups 2 and 4). After additional 24 hours, the fur was removed under full anaesthesia with isoflurane (IsoFlo® vet, Orion Pharma Animal Health, Copenhagen, Denmark), and the mice were placed onto the stage of the IVIS Spectrum (Caliper, LifeSciences, MA, USA) for fluorescence imaging. The following excitation and emission filters (Ex/Em) settings were applied: 675/720, 675/740, 675/760, 675/780, 660/605, 680/605, 700/605, 720/605, 740/605, 760/605, and 780/605. After in vivo imaging all animals were anesthetized by i.p. injections with a mixture of 0.5 mg/g body weight ketamine and 0.2 mg/g body weight xylazine (Ketaminol® Vet and Narcoxyl® Vet, Intervet, Skovlunde, Denmark), and the kidneys were dissected and subsequently washed thrice in PBS pH 7.4 before further scanning. All animals were finally sacrificed by cervical dislocation.

The acquired data quality was controlled using Living Image® 4.3.1 (Caliper Life Science, MA, USA) by determining if the acquired counts were above 600 and below 6 × 10^4^ raw counts in the specific image colour scale. Image correction was carried out by using adaptive fluorescence background subtraction in the data management software. We use the calibrated unit radiant efficiency instead of raw data counts to account for the excitation light projection on to the stage and the nonuniformity of that light projection in order to get a quantitative output. This was done to normalize the excitation lighting intensity per square area of the field of view. The unit that is utilized is therefore termed average radiant efficiency and is given as (photons/sec/cm^2^/sr)/(*μ*W/cm^2^).

### 2.10. Statistics

**Figure 2:**
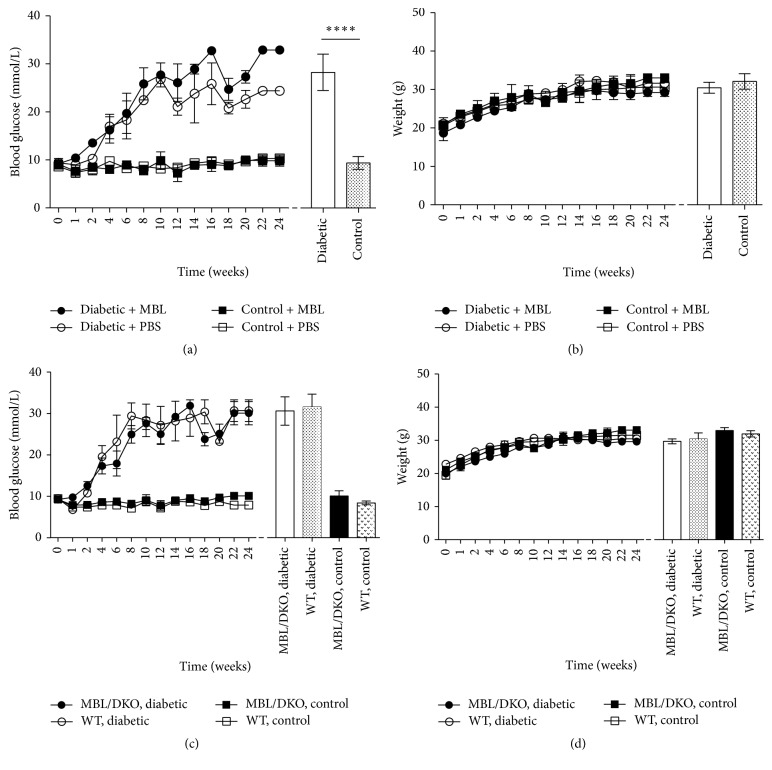
Blood glucose and weight gain of diabetic and nondiabetic control MBL/DKO and WT C57BL/6J mice. (a and b) Blood glucose and weight measurements for diabetic mice destined to be treated with rMBL AF680 (*n* = 6), diabetic mice destined to be treated with PBS (*n* = 6), control mice destined to be treated with rMBL AF680 (*n* = 6), and control mice destined to be treated with PBS (*n* = 6). (c and d). Comparison of the development of diabetes according to rising blood glucose levels and weight gain between diabetic (*n* = 6) and control (*n* = 6) C57BL/6J MBL/DKO mice and diabetic (*n* = 6) and control (*n* = 6) C57BL/6J WT mice. Statistical tests are given in the figure when significant, and comparisons are made between STZ treated and untreated groups at study end (^*∗∗∗∗*^*p* < 0.0001). Values are given in mean (dots) with SD (whiskers).

**Figure 3:**
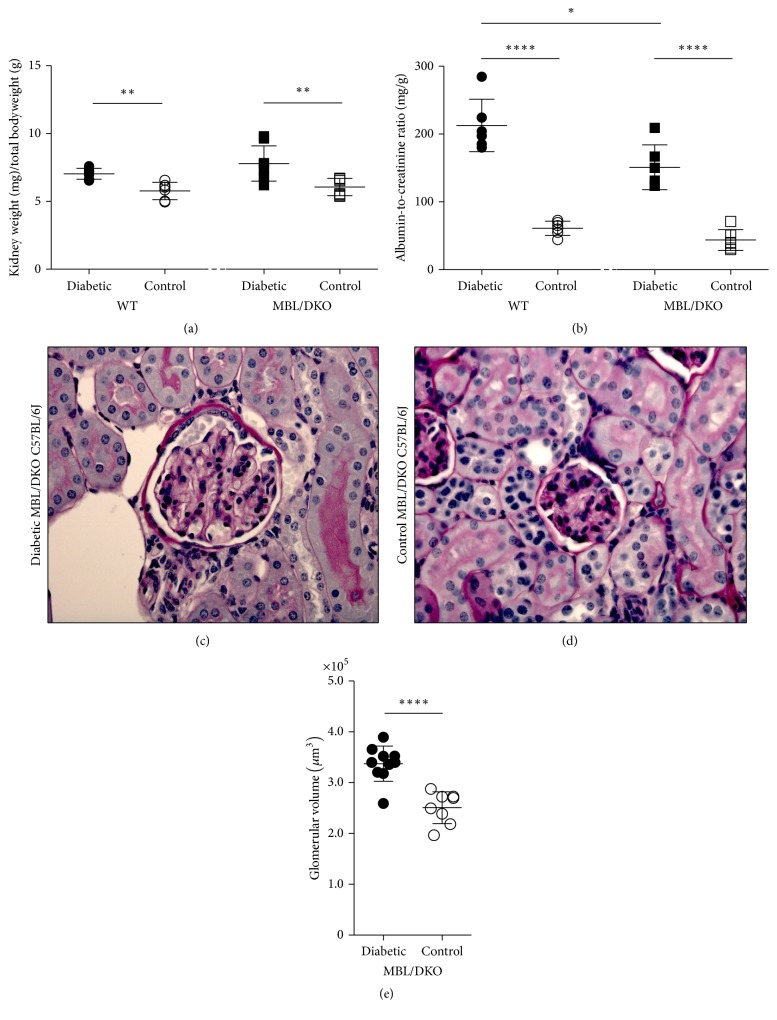
Kidney-to-body weight ratio, albumin-to-creatinine ratio, and kidney glomerulus estimation. (a) Kidney-to-body weight ratio of diabetic C57BL/6J WT mice (*n* = 6), nondiabetic control C57BL/6J WT mice (*n* = 6), diabetic C57BL/6J MBL/DKO mice (*n* = 6), and nondiabetic control C57BL/6J mice MBL/DKO (*n* = 6). (b) Albumin-to-creatinine ratio (AcR) measurements at study end for WT and MBL/DKO mice. (c and d) Light microscope representation of glomerulus from diabetic C57BL/6J MBL/DKO mice (c) and nondiabetic C57BL/6J MBL/DKO control mice (d). (e) Estimation of glomerulus volume in diabetic C57BL/6J MBL/DKO mice (*n* = 13) and nondiabetic control C57BL/6J MBL/DKO mice (*n* = 8). Statistical tests are indicated in figure when significant (^*∗*^*p* < 0.05, ^*∗∗*^*p* < 0.001, and ^*∗∗∗∗*^*p* < 0.0001). Data is presented as mean (solid line) with SDs (whiskers).

Student's *t*-test was used to compare diabetic and nondiabetic control groups from data in Figures 2 and 3. We used two-way ANOVA to test for interaction (*P*_int_) between diabetes status (diabetes/control) and MBL self-recognition (MBL/PBS), that is, investigating if diabetes would modify the potential self-recognition by MBL. Student's nonparametric two-tailed Mann–Whitney *t*-test was used to compare diabetic and nondiabetic control groups in the IVIS study. All data are expressed in mean with standard deviations (SD) with* N* indicating the number of observations or mice in each group.* p* values < 0.05 were considered significant. All statistical analysis for normality distribution were made using Stata 13 (StataCorp LP, College Station, TX, USA), while *t*-tests and two-way ANOVA analysis were made with GraphPad Prism 5 (GraphPad Software, Inc., La Jolla, CA, USA).

## 3. Results

### 3.1. Animal Characteristics: Blood Glucose Levels

BG levels did not differ between the prediabetic and the nondiabetic control C57BL/6J MBL/DKO mice groups at start of the study (*p* = 0.42). However, at study end the average BG levels were 28.4 mmol/L (CI: 24.3–32.7) in the diabetic group and 9.4 mmol/L (CI: 7.5–10.8) in the control group (*p* < 0.0001) (Figure 2(a)). When comparing BG levels of the STZ treated MBL/DKO animals with the STZ treated WT animals we found no difference between the two groups at start of the study (*p* = 0.95) (data not shown) or end of the study, 29.4 mmol/L (CI 26.0–33.0) and 30.3 mmol/L (CI: 27.1–33.5) (*p* = 0.63), respectively (Figure 2(c)). There was further no difference in the BG levels between the two nondiabetic groups at the start (*p* = 0.62) (data not shown) or end of the study, 9.7 mmol/L (CI: 8.4–11) and 8.1 mmol/L (CI: 7.6–8.5) (*p* = 0.07), respectively (Figure 2(c)).

### 3.2. Animal Characteristics: Body Weight

Total BW did not differ between the diabetic and nondiabetic control groups at neither start (*p* = 0.31) (data not shown) or end of the study, 30.5 g (CI: 29.0–32.0) and 32.15 (CI: 30.5–34.25) (*p* = 0.13), respectively (Figure 2(b)). When comparing initial BW between the WT and MBL/DKO strains treated or left untreated with STZ we also found no difference (*p* = 0.16 and *p* = 0.24, resp.) (data not shown). Likewise, the BW at the end of the study did not differ for the corresponding diabetes groups, 29.5 g (CI: 28.7–30.3) and 30.2 g (CI: 28.3–32.1) (*p* = 0.39) (Figure 2(d)). Similarly, we found no difference when comparing initial BW between the two untreated strains (data not shown) (*p* = 0.24). Final BW for the corresponding untreated groups did likewise not differ, 32.75 g (CI: 31.8–33.7) and 31.7 g (CI: 30.8–32.7), respectively (*p* = 0.078) (Figure 2(d)).

### 3.3. Diabetes-Induced Kidney Changes

To verify that the STZ-induced mice indeed exhibited diabetes-induced kidney changes we further investigated the kidney morphology. When comparing the kidney weights of the diabetic WT mice to nondiabetic control WT mice we observed that diabetes induced a 22% increase in the kidney-to-body weight ratio, 7.1 mg/g (CI: 6.6–7.4) and 5.9 mg/g (CI: 5.0–6.3), respectively (*p* = 0.002) (Figure 3(a)). Furthermore, there was a 29% increase in the kidney weight of the diabetic MBL/DKO mice as compared to nondiabetic control MBL/DKO mice, 7,7 mg/g (CI: 6.8–9.2) and 6.0 mg/g (CI: 5.4–6.7), respectively (*p* = 0.004). This is consistent with previous observations [8]. In contrast to previous observations regarding morphological changes in the kidney due to diabetes there was no difference between diabetic WT and diabetic MBL/DKO mice (*p* = 0.28). The albumin-to-creatinine ratios were significantly elevated in the diabetic mice in both the WT group, 212.7 mg/g (CI: 172.2–253.2), as compared to the nondiabetic control WT mice, 61.2 mg/g (CI: 50.2–72.1) (*p* < 0.0001), and the diabetic MBL/DKO group, 150.9 mg/g (CI: 116.1–185.7) as compared to nondiabetic control MBL/DKO mice, 43.9 mg/g (CI: 27.7–60.0) (*p* < 0.0001), respectively (Figure 3(b)). Diabetic WT mice had a significantly higher AcR as compared with the diabetic MBL/DKO mice (*p* = 0.02). Also as previously observed the total glomerulus volume increased by 52% in the diabetic MBL/DKO group as compared to the nondiabetic control MBL/DKO group (*p* < 0.0001) (Figures 3(c)–3(e)) [8]. As expected, urine production was elevated in the diabetic mice group as compared to control mice (data not shown).

### 3.4. Test of Function of the AF680 Labelled rMBL and Protein Staining

rMBL and AF680 fluorophore was mixed at a weight ratio of 1 : 20. This mounted to a degree of labelling (DOL) of approximately one AF680 fluorophore per rMBL polypeptide chain (see Section 2.6). MBL is an oligomeric molecule build from identical subunits that each contains three polypeptide chains (forming a collagen-like region). The used rMBL preparation consists largely of pentamers, hexamers, and heptamers of such subunits and has thus 15–21 AF680 fluorophores per rMBL molecule.

**Figure 4:**
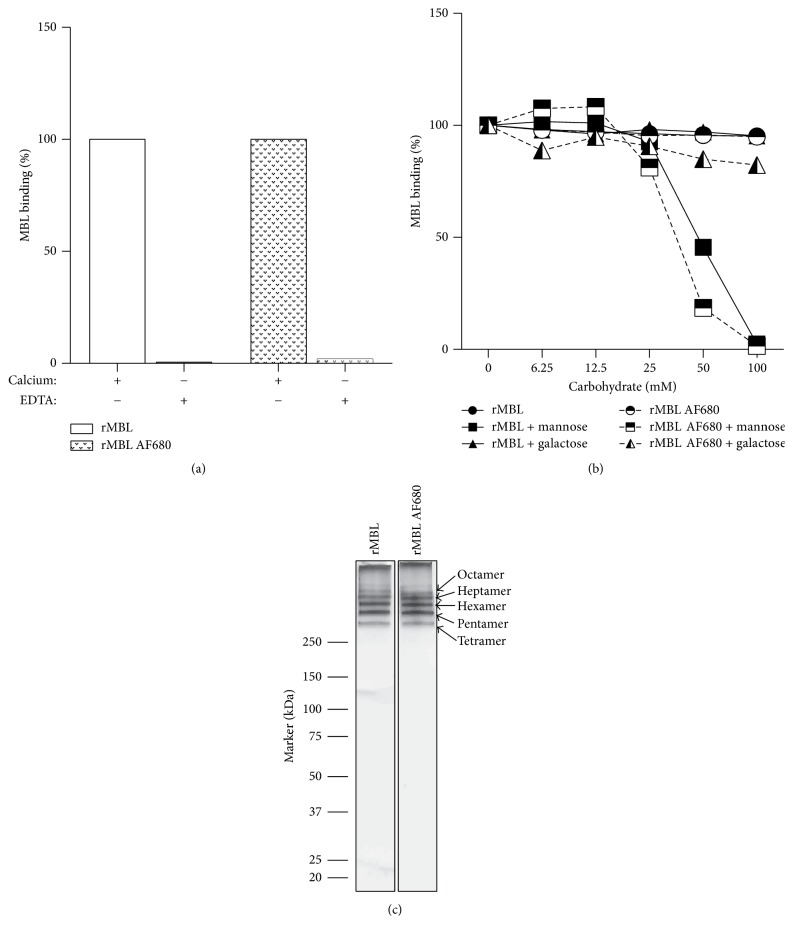
Analysis of retained functionality of AF680 labelled rMBL. (a) rMBL and rMBL labelled with AF680 were analysed for retained binding activity onto a surface of mannan in presence of calcium or EDTA. Values measured in calcium buffer were defined as 100% and subsequent samples were calculated according to these. (b) AF680 labelled rMBL and unlabelled rMBL were analysed for binding activity in the presence of mannose or galactose. Values obtained in the presence of galactose were defined as 100% and subsequent samples were calculated according to these. (c) SDS-PAGE followed by silver staining of rMBL and rMBL AF680 with arrows indicating the distribution of rMBL oligomers. Molecular marker is given in kDa. All values are given in percentage (%) of bound rMBL with values obtained in the presence of galactose defined as 100% bound and subsequent samples calculated according to these. The experiments were repeated twice with similar results.

The labelled rMBL was tested for retained functionality in a solid-phase ligand-binding TRIFMA assay for MBL in two different setups investigating the calcium-dependent binding and sugar specificity (Figures 4(a) and 4(b)). The labelled MBL had retained the ability to bind in a calcium-dependent manner to mannan-coated surfaces (and could thus be inhibited by EDTA). Furthermore, regarding incubation with increasing concentration of the various carbohydrates used as inhibitors, we observed a dose-dependent inhibition of both labelled and unlabelled rMBL with mannose but not with galactose, which corresponds to previous observations (Figure 4(b)). When subjecting preparations of labelled and unlabelled rMBL to SDS-PAGE separation we observed no visible difference in oligomer distribution between these (Figure 4(c)). We thus concluded that labelling did not alter the functionality of rMBL.

### 3.5. In Vivo Fluorescence Imaging of AF680 Labelled rMBL Deposition in the Diabetic Kidney

**Figure 5:**
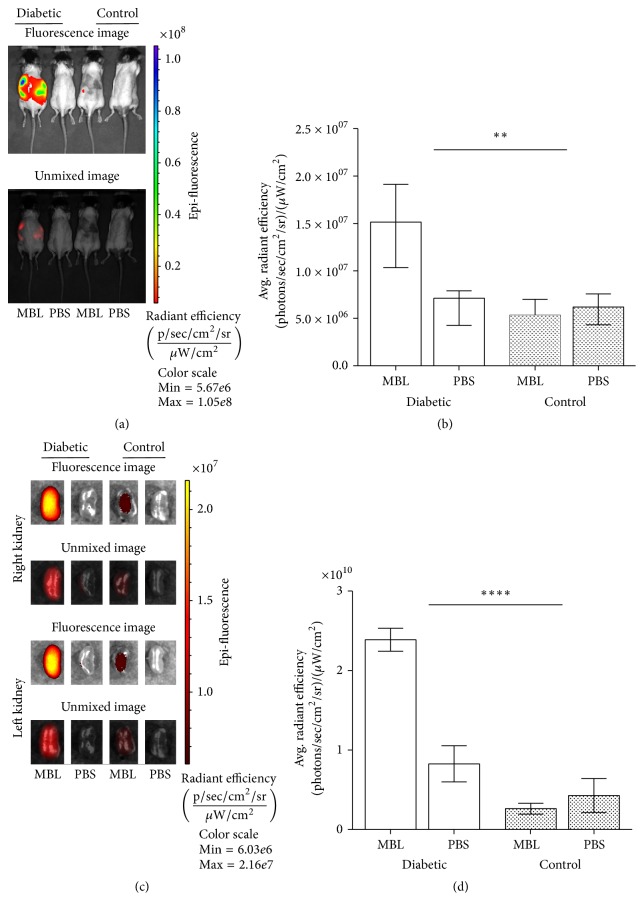
In vivo and ex vivo detection of AF680 labelled rMBL 24 hours postinjection. (a) Fluorescent and spectral unmixing images representing one mouse in each group exhibiting distribution of labelled rMBL 24 hours postinjection (dorsal view). (b) Region of interest (ROI) quantification of detected fluorescent signal from diabetic C57BL/6J MBL/DKO mice + AF680 rMBL (*n* = 6), diabetic mice + PBS (*n* = 6), nondiabetic mice + AF680 rMBL (*n* = 6), and nondiabetic mice + PBS (*n* = 6) given in average radiant efficiency. (c) Ex vivo fluorescent and spectral unmixing images representation of the left and right kidneys from one mouse in each group. (d) ROI quantification of detected fluorescence in the right and left kidneys of mice in each group as in (b) with values given in average radiant efficiency. Scale bars are given in radiant efficiency. Statistical tests are indicated in the figure when significant (^*∗∗*^*p* < 0.001, ^*∗∗∗∗*^*p* < 0.0001). The experiment was repeated five times with comparable results.

To determine the distribution of labelled rMBL during late diabetes-mediated vascular complications in the kidney, we performed in vivo imaging of whole mice followed by ex vivo imaging of the dissected kidneys. We removed the fur before imaging to minimize autofluorescence interference. Only MBL/DKO animals were used for these experiments to avoid biases from endogenous MBL. Fluorescence imaging with the IVIS Spectrum system has the advantage of imaging the fluorescence of the whole-body of the mouse even though the spatial precision of this technique is rather low. The individual mouse exhibits a variable degree of autofluorescence with no clear distinction between the fluorescence coming from the different organs (Figure 5(a)). This was mainly caused by variation in pigmentation, fur colouring, animal size, food intake, and so on resulting in inconvenient background fluorescence. However, adaptive background fluorescence subtraction provided a more detailed distinction between control and diabetic animals given either MBL or PBS. When quantifying the obtained signal, the in vivo imaging suggested that diabetes significantly modified the effect of MBL treatment; that is, we observed an effect of diabetes on the autoreactivity of MBL (MBL × Dia) (*P*_int_ = 0.002). We furthermore tested for difference between treatments with MBL and PBS in the nondiabetic control group and observed no difference (*p* = 0.93) (Figures 5(a) and 5(b)). Ex vivo imaging was subsequently performed immediately after sacrificing the mice. Results obtained from the ex vivo imaging of the kidneys further showed specific accumulation of MBL in the kidneys. Thus, we concluded that diabetes significantly modified the effect of MBL self-recognition (MBL × Dia) (*P*_int_ < 0.0001) (Figures 5(c) and 5(d)). There was again no difference between treatments with MBL or PBS in the nondiabetic control group (*p* = 0.47) (Figures 5(c) and 5(d)). These results strongly indicate that MBL accumulates in the diabetic kidney. The results are summarized in Table 1.

## 4. Discussion

Several studies have suggested that the complement system may be involved in the pathogenesis of diabetic nephropathy. In the present report, we show for the first time in an in vivo model that MBL has increased autoreactivity as a response to diabetes. Our findings shed new light on the role MBL and the complement system and suggest a therapeutic potential of complement inhibition in diabetic vascular complications.

It has previously been shown that MBL-mediated complement activation is associated with IgA nephropathy and Henoch-Schonlein purpura nephritis [17, 19], and our group have previously shown that there is a clear association between high serum levels of MBL and increased risk of developing microalbuminuria and diabetic nephropathy [7, 26, 27]. Furthermore, serum levels of MBL are significantly elevated in patients with type 1 diabetes when comparing to healthy individuals [5, 28]. Furthermore, our group have recently described that all-cause mortality is strongly associated with MBL genotype and MBL concentration in type 1 diabetes [29].

The efficiency of the innate immune system depends on the ability to distinguish the host from microorganisms and thus self from nonself. The lectin pathway of the complement system is initiated when MBL recognizes and binds evolutionary conserved pathogen-associated molecular patterns (PAMPs) and initiates the complement cascade [30]. These structures are typically found on invading microorganisms of both bacteria and viruses, thus discriminating self from nonself [15]. However, it is hypothesized that MBL may initiate the lectin pathway by recognizing damage- or danger-associated molecular patterns (DAMPs) and altered host-associated molecular patterns (HAMPs), which may be present on damaged or altered host tissue or cells [15]. Diabetes has been shown to be associated with increased unspecific glycations of host proteins, lipids, endothelia, and cells [20]. Based on our new findings it is highly plausible that MBL is able to recognize such diabetes-induced DAMPs and/or HAMPs.

In the present study, we have for the first time shown that MBL accumulates in the kidney during late diabetic nephropathy. The main objective of this study was to investigate the possible autoreactivity activity of MBL during diabetic nephropathy. The MBL double-knockout mouse model on the C57BL/6J background was chosen because we wanted to be able to fully exploit any diabetes-induced neoepitopes generated in the kidneys. We thus eliminated any endogenous MBL competition by the use of the MBL KO mice.

The STZ-model of type 1 diabetes depends on the active uptake of the glucose composition of STZ via the GLUT2 transmembrane protein expressed primarily in liver and pancreatic *β*-cells, which initiates DNA repair in the cell via NAD^+^ [31]. This depletes the cell of NAD+ and ultimately inhibits metabolism and causes cell death. The STZ-model of type 1 diabetes was further validated by the diabetes-induced kidney hypertrophy and albumin excretion found in treated C57BL/6J mice. Both the kidney-to-body weight ratio and the AcR were significantly elevated in diabetic mice indicating kidney damage. When further investigating the kidney morphology we observed a significant enlargement of the glomerular volume in diabetic mice compared to healthy mice, thus confirming development of diabetic nephropathy.

When analysing the in vivo fluorescence imaging after injection of labelled MBL we observed a fluorescence pattern located in the internal organs including the kidneys. However, a disadvantage of the whole-body imaging is that fluorophore excitation gets scattered in all directions throughout the body, thus rendering detection and quantification of the specific origin difficult. Furthermore, the C57BL6/J mice exhibit pigmentation that exhibits greater autofluorescence than the preferred nude mouse model for studying fluorescence in vivo. However, this was not possible with the desired genetic MBL/DKO background. A more lucid result that MBL indeed was accumulating in the kidneys was acquired, when the data obtained by the ex vivo imaging of the kidneys was analysed. The ex vivo imaging results were obtained from several independent experimental studies. It is conceivable to speculate that the total number of MBL molecules binding in the glycated tissue in the kidney may be low, thus producing a poor signal-to-noise ratio. However, these new findings support our hypothesis that MBL is bound and accumulates in the diabetic kidney during the development of diabetic kidney damage.

In conclusion, we here show for the first time in vivo and ex vivo data that supports the hypothesis that MBL accumulates in the diabetic kidney. This is the first direct evidence that autoreactivity of MBL indeed may play a role in the pathophysiology of diabetic nephropathy. The present report also suggests that a labelled form of MBL could potentially be used as a tool for detecting altered glycosylation of structures within the body. Future studies are needed to further investigate the detailed consequences of autoactivation of the complement system via the lectin pathway and MBL in diabetes.

## Figures and Tables

**Table 1:** Summarized data and statistical information of in vivo and ex vivo imaging of diabetic and nondiabetic mice.

	Diabetic		Control		Two-way ANOVA^*∗*^
	MBL (*n* = 6)	PBS (*n* = 6)	MBL (*n* = 6)	PBS (*n* = 6)	
In vivo	1.5 × 10^7^ (9.9 × 10^6^–1.9 × 10^7^)	6.8 × 10^6^ (4.5 × 10^6^–8.5 × 10^6^)	5.8 × 10^6^ (3.2 × 10^6^–8.4 × 10^6^)	5.9 × 10^6^ (4.0 × 10^6^–7.4 × 10^6^)	*p* = 0.0021

Ex vivo (kidney)	2.4 × 10^10^ (2.0 × 10^10^–2.8 × 10^10^)	8.2 × 10^9^ (2.4 × 10^9^–1.4 × 10^10^)	2,6 × 10^9^ (8.0 × 10^8^–4.3 × 10^9^)	4.3 × 10^9^ (1.2 × 10^9^–9.8 × 10^9^)	*p* < 0.0001

Values are given in mean radiant efficiency with 95% confidence intervals.

^*∗*^ *p* values for the hypothesis of “no interaction between MBL and diabetes.”
